# Mr. Paget’s Report Continued

**Published:** 1844-04-20

**Authors:** 


### MR. PAGET'S REPORT CONTINUED.

#### NERVOUS SYSTEM.

*Minute Structure.* Remak says that on the axes of each of the larger primitive tubes of the abdominal nervous cord of the River Cray Fish (*astacus fluviatilis,*) there is, in the recent state, a winding bundle of extremely delicate fibres occupying one-third or one-fourth of the whole diameter of the tube. The fibres of this *central fasciculus* are smooth, parallel, without branches or anastomoses, and less than 1-5000 of an inch thick. They may be seen distinctly when the tubuli are injured; some of them often protrude from the broken extremity. They are found however, only in tubules from 1-60 to 1-30 of a line in diameter; smaller tubules than these either appear translucent or contain a fine granular substance, and none but the smaller tubules, such as these, are found in the nerves, and nervous trunks near the abdominal cord. The spaces between the central fasciculi and the walls of the larger tubules are filled by a clear, colourless fluid. The relation of the central fasciculus in the large tubules of the nervous cord to the central substance of ordinary nerves (the *primitive band* of Remak) is uncertain.

*Repair and union of nerves.* Dr. Bidder, of Dorpat, has made several experiments to determine whether nervous filaments of originally different functions can be made to unite. He experimented on the lingual and hypoglossal nerves of dogs, but the results were inconclusive. They, tended, however, to prove that such a union does not take place: for in several of the cases the connected portion of the two trunks were found, on subsequent examination, disparted, and each had united again with that portion of itself from which it had been separated. It was found that a sufficient union for the restoration of function can take place in three or four months, although a portion of a nervous trunk eight lines in length has been completely removed.

*Reflex action.* Some evidence in favour of the view that the nerves of the excitomotory system form a system distinct from those conveying sensation and volition, is afforded by the investigations of Mr. Newport. He finds that in the myriapoda the fibres which correspond to the true spinal cord in vertebrata are distinct from those connected with the cephalic ganglia. They form part of the cord in the intervals between the abdominal ganglia, and may be traced from the periphery into the several ganglionic centres from which they pass backwards along the cord until they arrive at the next ganglion, from which they pass again to the surface of the body. Now there is reason to believe that the ganglia are not sensitive: for the reflex acts, which are repeatedly performed after the removal of the head or destruction of the central ganglia, are performed without any appearance of volition being exercised in them, and always in one and the same manner.

*Influence of the nervous centres.* Professor Volkmann, one of the most accurate experimenters of modern times, has occupied himself in testing the value of those experiments which are supposed to prove the direct influence of the central nervous organs upon the movement of the viscera.

With regard to the part of the centres on which the movements of the heart depend,—Volkmann shows that in fresh-slain animals the movements of the heart are so completely irregular, even when left to themselves—in one half minute hurried, in the next retarded, then stopping for one or more minutes and then of themselves going on again—that it is impossible to determine the influence of any supposed excitant of the brain or spinal cord. From a great number of experiments, very carefully conducted, no fixed result could be arrived at except this, that the existence of any direct influence exerted on the heart by irritating the nervous centres is as yet altogether doubtful.

He has come to the same conclusion, upon equally good negative evidence, in regard to the effects supposed to be produced on the motions of the stomach and intestines by irritating the brain and cord. He could find no such influence exerted. The motions of the alimentary canal often entirely cease for a long time, and are then of themselves renewed; but when once they have entirely ceased, no irritation of the nervous centres can reproduce them, although it is certain that after the canal ceases to move, the nervous centres are still irritable. He peremptorily denies Budge's experiment in which he believed that though the peritoneum of the abdominal walls was left, the intestines moved when the central organs were irritated, and ridicules the *active inflation* of the stomach, which Budge supposed to be thus produced. With equal positiveness he denies the truth of Budge's statements respecting the elevation and expansion of the testicle when a part of the cerebellum is irritated. In repeated trials he could produce no such effects as Budge reports, in either the digestive canal or the testes. His conclusion is, "I am far from denying that the central organs exercise an influence on the motions of the viscera, for pathological observations make that certain. But they do not prove that this influence is a direct one; and experiments on living or fresh-slain animals prove it still less."

*Brain.* In a very valuable contribution to the statistics regarding the weights of organs—of which, however, the greater part can as yet serve only to add to the necessary heap of evidence—Dr. John Reid has made it probable—1. That the cerebellum does not attain its maximum weight at seven or a few more years of age, though it does attain it sooner than the other organs, and the size of the whole brain, in proportion to the entire body, is greater in the child than in the adult. 2. That the average weight of the cerebellum compared with that of the whole brain, is a little greater in the female than in the male. 3. That though the male brain is on the average heavier than the female, yet in proportion to the weight of the whole body, it is rather less heavy. 4. That the brain does not in emaciation diminish in the same proportion as the rest of the body does.

M. Parchappe has shown from his measurements and weighings, that in regard to *the size of the head*—1, that of males is to that of females as 16,128: 15,294; 2, it increases gradually to the sixtieth year, chiefly through enlargement of the frontal sinuses, and after that time diminishes; 3, it is in some measure proportioned to the stature. And in regard to the *size of the brain*—1, that the male is to the female brain, on an average, as 156; 125, and that in weight they have about the same proportion; 2, that it increases to the fortieth year, and then decreases to the seventieth; 3, that it bears some proportion to the stature; 4, that the intellect is not absolutely proportioned to the size of the brain, but is proportioned to the size of the hemispheres, and especially to the extent of their surfaces.

Dr. George Burrows having repeated the experiments of Dr. Kellie and performed others, has shown, in opposition to the opinions commonly entertained, 1st, that the brains of animals bled to death are deprived of their blood, and rendered pale and anæmic; 2, that the quantity of blood in the head is greatly affected by posture and gravitation; 3, that in death by apnœa there is intense congestion of the cerebral vessels. And from these facts and from several considerations he deduces that the opinion that the quantity of blood within the cranium is at all times the same is untrue. Admitting that the total contents of the cranium must be at all times nearly the same, the cerebro-spinal fluid is rapidly removable from one site to another, and capable of being altogether removed by absorption; so that this fluid may be regarded as supplemental to the other contents of the cranium—at one time giving place to the increased quantity of blood, at another making up for the deficiency of blood in the vessels, and in the same manner varying according to the actual quantity of nervous substance.

*Spinal cord.* From Dr. Knox we have a description of the spinal arachnoid, maintaining that the account usually given of it and of the absence of any regular communication between its cavity and that of the cerebral ventricles is correct, in opposition to the descriptions of Dr. Sharpey and Mr. Ellis.

Drs. Stilling and Wallach deny the existence of globules in the gray matter of the spinal cord, and say that those which have been held to be globules are fragments of divided nerve-tubes. These tubes of the gray matter they describe as differing from those of the white in being of less diameter, having thinner external walls, and being differently coloured. The course of some of them is longitudinal; that of others transverse, and these are continued into the white substance of the cord, crossing the direction of its fibres, but never uniting with them. The filaments of the roots of the nerves are continuous, not with the white filaments or tubules of the cord, but with these its transverse gray filaments.

In some "Additional experiments on the spinal marrow," Dr. Van Deen mentions two which he has frequently repeated in the presence of competent judges, to prove that the nervous fibrils of the limbs of the frog do not proceed to the brain, but terminate in the spinal marrow. In the first experiment the whole spinal marrow of a frog is exposed, and *all* the roots of all the nerves which go to the fore-legs and abdomen are cut on both sides; the marrow is then divided a little above the place where the nerves of the fore-legs are cut through, and its divided end being gently raised, a portion of glass or paper is pushed under it; but this is done only for the convenience of the further cutting. If now small portions of the spinal marrow are cut off successively from above downwards with great care and without shaking, no muscular movements are excited in the hind-legs; and the sections may be continued to within a little of the place at which the first lumbar nerve leaves the spinal marrow. It is on cutting this part that one first sees muscular motions in the upper part of the thigh; and as one goes on cutting lower down they ensue in both the hind feet.

In the second experiment all the roots of all the nerves of the hind legs are first cut on both sides of the cord, and a portion of paper being put under the lower end of the cord, pieces of the cord are cut off in succession from below upwards. No signs of pain are induced, nor (even when the animal is beheaded) is any motion of the fore-legs excited, till one comes to that part of the cord from which the undivided roots are given off.

These two experiments prove, says the author, 1st, that the primitive fibrils do *not* pass through the spinal cord to the brain, since, if that were the case, every division of the cord in the first experiment must produce motion in the hind-legs, and every division in the second must have excited pain, or, at least, some motion of the fore-legs; and 2dly, that the spinal marrow is not capable of propagating any irritation communicated to it to a great distance through itself, unless nerves are connected with it.

An interesting case bearing upon the physiology of the several columns of the cord is related by Dr. Webster. There was complete loss of voluntary motion in the trunk and limbs, with retention of natural sensibility in them, and active reflex movements, in consequence of softening of the whole thickness of the middle part of the cervical portion of the spinal cord. The case is inexplicable in the present state of knowledge, unless we believe that Van Deen's experiments are conclusive, which seemed to prove that the gray matter of the cord can, generally speaking, convey centripetal impressions to the brain, but not centrifugal impressions from it, and, besides, suppose that in this case the morbid change of the gray matter was not so complete as wholly to interrupt its functions. There are other cases sufficient to prove that considerable degrees of softening and other changes of structure of the cord may exist without complete loss of function.

Mr. W. F. Barlow has published some good remarks on the influence of the impressions from sudden changes of temperature in producing reflex movements.

*Particular nerves. Optic.* Professor Erdl considers that he has traced the fibres of the optic nerves through the following long course: From the optic tract they diverge and expand in the substance of the thalami, then again converge towards the anterior part of the thalami, and unite into a cord distinguished by its white colour, which descends into the corpora albicantia, forms a loop in them, and then turning upwards and forwards ascends through the anterior crura into the body of the fornix. From the fornix the fibres are continued into its posterior crura, and into the corpora fimbriata, in which they descend into the pes hippocampi on each side, whence again they ascend in the tapetum to the posterior part of the corpus callosum, in which the fibres of the two sides again unite. He describes also the generally admitted fibres passing from one retina to the other through the anterior part of the optic commissure; and he supposes that the peripheral ends of these fibres are connected with those of the fibres whose course is described above, so as to form a kind of closed system or nervous ring.

*Third nerve.* Dr. Fäsebeck of Brunswick, describes a branch of the superior division of the third nerve, which is given off soon after that nerve enters the orbit, passes between the superior and external recti muscles of the eye, and penetrates the external rectus. He describes also a branch one-eighth of a line in thickness, going from the otic ganglion into the spheroidal sinus, another going from it to the vidian, and a third going to the tensor palati muscle.

*Facial nerve.* It was known that in paralysis of the facial nerve of one side, the uvula was commonly drawn to the opposite side, and this was supposed to indicate that the facial is the motor nerve of the palate. Some doubt was thrown upon the conclusion by M. Debrou, who showed that the uvula in many persons was naturally not suspended in the middle line. But this objection has been removed by M. Diday, who has observed a case in which the uvula was drawn to the opposite side while the paralysis of one nerve lasted, but gained its straight position when the paralysis ceased. It seems probable, therefore, that at the junction of the superior petrous branch of the vidian with the facial, branches are sent from (not to) the latter which go to the spheno-palatine ganglion, and thence through the posterior palatine nerves to the soft palate, as Soemmering believed.

*Corda tympani.* Dr. Guarini states that he can demonstrate visibly that the corda tympani comes off as a distinct branch from the facial nerve, without any communication with the vidian. He gives the following reasons for believing that through the corda tympani the facial has a motor influence on the tongue. His experiments were often repeated before Panizza and others. 1. When the hypoglossal nerve is galvanized the tongue is moved convulsively forwards and backwards and upwards and downwards, but the fibres of the middle portion are quiet. 2. When the trigeminus is galvanized the tongue never moves. 3. When the facial is galvanized the tongue moves quickly upwards and downwards, and there is a kind of vermiform motion of its middle part. The first movement depends on the styloglossi muscles, which have a distinct branch from the facial; the second on the linguales, to which branches can be traced from each corda tympani. 4. After dividing the hypoglossal nerve the movements of the styloglossus and lingualis alone continue, and these may be excited by galvanizing the facial. But the vermiform motion does not continue after (in the same case) the corda tympani is destroyed.

*Nervous vagus* and *nervous accessorius.* Mr. Spence has contributed a fact of great importance to the reconciling of the contrary statements respecting the motor functions of the pharyngeal and inferior laryngeal branches of the vagus nerve. He has traced a filament (distinguished from the rest of the vagus by its white colour,) which, arising from the groove between the olivary and restiform bodies, passes along the course of the vagus trunk, but goes over without joining the superior ganglion, and does not join the vagus trunk till just above the inferior ganglion. At this point of junction it is also joined by the internal branch of the accessorius, and from the junction of the two the pharyngeal branch of the vagus is given off. The conjoined white cord then descending with the vagus, seems to pass principally into the recurrent nerve, and probably sends filaments into the œsophageal branches.

Mr. Spence, whose dissections agree nearly with those of Bendz, proposes for the separated white cord of the vagus the name of the motor column of the vagus, (motor *root* would perhaps be better,) and likens its arrangement to that of the motor root of the fifth nerve, passing under the Gasserian ganglion, and joining the trunk beyond it. The pharyngeal branch of the vagus and the recurrent laryngeal being thus given off from the internal branch of the accessorius and from a motor root of the vagus, their purely motor functions are sufficiently accounted for; and the experiments of Dr. John Reid, in which irritation of the roots of the vagus nerve produced movements of the larynx, are explained. It is still, however, not clearly explained why his experiments of irritating the accessorius within the skull did not (as those of M. Longet did,) produce movements of the same organ. It is possible that all the filaments of the nerves were not implicated in the irritation.

A confirmation amounting almost to proof of this view of the influence of the nervus accessorius in the movements of the human larynx, is afforded by the fact, that according to Professor W. Vrolik, the internal branch of the nervus accessorius in the chimpansé does not join the vagus, but goes at once and separately to the larynx, while the external branch is distributed almost exclusively to the trapezius. And other facts confirmatory of the same view are supplied by the experiments of Signor Morganti; which also tend to show that the external branch of the accessorius contains the fibres which have their origin lowest down on the cord, while the internal branch contains those which arise higher up, just below the vagus, of which the author considers the accessorius to be the anterior root.

*Spinal nerves.* Mr. Viner Ellis has described minutely the arrangement of the posterior branches of the spinal nerves. Within the extent of the multifidus spinæ muscle, including therefore all the spinal nerves, except the suboccipital and the last two sacral and the coccygeal, all the posterior divisions of the spinal nerves have an external and an internal branch. Of the cervical nerves the external branches supply the cervicalis ascendens, transversalis colli, and trachelo-mastoid muscles; the internal and larger branches supply the multifidus spinæ, semi and interspinales, and those of the four highest give off cutaneous branches. Of the posterior divisions of the dorsal nerves the internal branches penetrate the multifidus spinæ and semispinalis, and give cutaneous nerves from the six upper; the external branches enter the erector spinæ and levatores costarum, and cutaneous portions spring from about the six lower. In the lumbar nerves the internal branches of the posterior divisions end in the multifidus spinæ, and the external branches, giving cutaneous nerves from the three upper, terminate in the erector spinæ. In the three upper sacral nerves the internal branches enter the multifidus spinæ, the external and larger become cutaneous after uniting by anastomotic arches with each other and with the external branch of the last lumbar and fourth sacral nerves.

*Sympathetic nerves.* Dr. A. Von Walther has made experiments in which (after exposing them from behind, he has divided upon the front of the spine, near the sacrum and close to the aorta, some four or seven filaments passing from one of the trunks of the sympathetic nerve to the ischiatic plexus. (The mode of separation is acurately described.) The nearly constant result was, that after two days the capillary circulation on the operated side became more rapid, the capillaries smaller, and the blood-corpuscles in them disproportionately few. This lasted till about the fifth day; then for a day the circulation became natural, and then it became slower and pulsatile, and gradually ceased. The blood-corpuscles accumulated in the larger vessels in spots, exudation took place, and the membrane of the web became soft and rotten.

With one exception in many experiments these changes occurred only on the foot of the injured side; yet, ingenious as they are, there must be much doubt as to the sufficiency of these experiments to prove the influence of these sympathetic nerves upon the irritation of the limb. The mere injury of the other nerves of that side would produce some effect, especially in animals tied down for fourteen or more days.

Dr. Fäsebeck finds a sublingual ganglion between the mylohoideus muscle and the sublingual gland, about two lines from the lower border of the latter. It is a round, flat, grayish-red swelling, about a line in diameter, and receives branches from the lingual branch of the fifth, from the corda tympani, and from the filaments of the plexus around the sublingual artery. There proceed from its anterior and inferior part six branches, which penetrate the sublingual gland, and of which one accompanies the duct. He describes also six ganglia, each from one to three lines in diameter, between the lower part of the trachea and œsophagus, and between the latter and the spine, formed on branches of the sympathetic, vagus, and recurrent nerves, and giving filaments to the cardiac plexus, aorta, pulmonary artery, thoracic duct, superior cava, trachea, œsophagus, and pericardium.

